# Highly stretchable transparent Ag nanowire-polyurethane hybrid bilayer electrodes for multifunctional applications

**DOI:** 10.1080/14686996.2025.2528595

**Published:** 2025-07-04

**Authors:** Fang Luo, Seo-Yun Choi, Yewon Lee, Jun-Hyeok Kang, Joon Jang, Hyun-Jung Jung, Seungmin Lee, Ji-Yoon Chae, Han-Ki Kim

**Affiliations:** School of Advanced Materials Science and Engineering, Sungkyunkwan University, Suwon-si, Kyunggi-do, Republic of Korea

**Keywords:** Ag nanowire, polyurethane, mixed electrode, bilayer, heater, strain sensor, interconnector

## Abstract

We developed an Ag nanowire-polyurethane (AgNW-PU) mixed electrode on a PU substrate with an optimized bilayer structure for highly stretchable and wearable strain sensors. In the AgNW-PU mixed composite, PU functioned as a stretchable matrix, preserving the high conductivity and transparency of the AgNW network even under applied mechanical stress. The AgNW-rich bottom layer (25:1) provided an effective conduction path, whereas the PU-rich top layer provided mechanical support and elasticity, improving the durability of the electrode under repeated stretching and bending cycles. With the optimized bilayer (AgNW-PU 100:1/25:1), the AgNW-PU bilayer electrode exhibited a low sheet resistance of 26.3 Ω/square and a high transparency of 86.4%. Compared with the AgNW-PU single-layer electrode, the bilayer electrode exhibited superior stretchability, as confirmed by various applications, such as heater devices, strain sensors, and interconnectors. An optimized AgNW-PU bilayer electrode exhibited heat generation of 90°C with 7 V applied even after 15% stretching. The gauge factor of the optimized electrode increased from 8 to 11.2 even as the bending degree increased from 30° to 90°. The AgNW-PU bilayer electrode also demonstrated potential as a stretchable interconnector for various next-generation electronic applications.

## IMPACT STATEMENT

Regarding the AgNW-PU bilayer electrodes for flexible and stretchable electronic devices, we highlight two main reasons for the novelty and significance of our research. First, we systematically investigated a bilayer structure featuring a PU-rich top layer and an AgNW-rich bottom layer, and found that this configuration significantly enhanced the mechanical stability and conductivity of the electrode under tensile and bending strains. Second, we confirmed that the AgNW-PU structure achieves an excellent balance between high optical transparency and low sheet resistance, maintaining outstanding electrical performance even after repeated deformations. We believe that the structural design and strain-response mechanism revealed in this study provide a theoretical basis for the development of high-performance stretchable electrodes, with potential for practical applications in next-generation flexible displays, wearable sensors, and electronic skin.

## GRAPHIC ABSTRACT

**Figure:**
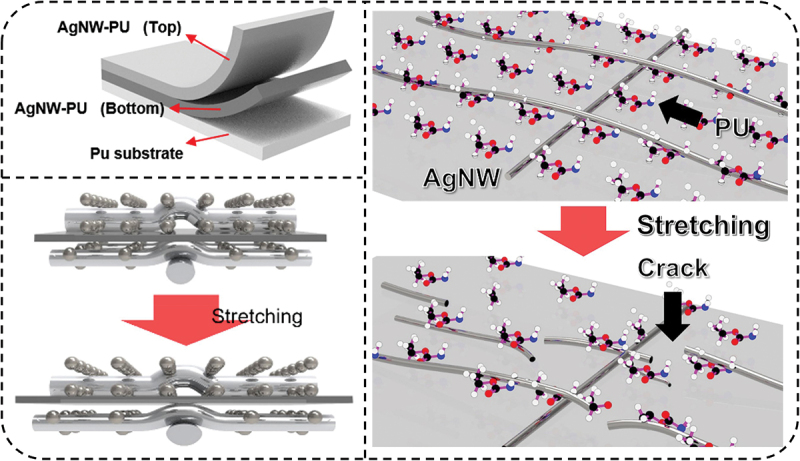

## 1. Introduction

The development of high-performance stretchable transparent conductive electrodes (STCEs) has become a critical research frontier in flexible electronics, as these materials must simultaneously satisfy stringent requirements for mechanical stretchability, electrical conductivity, and optical transparency [1–6]. While conventional indium tin oxide (ITO) electrodes suffer from brittle failure under mechanical deformation, emerging materials such as graphene, carbon nanotubes (CNTs), and elastomer (e.g. polydimethylsiloxane, and Ecoflex)-based composites have shown promise for STCE applications [7–14]. Nevertheless, graphene electrodes suffer from low stretchability owing to the brittleness of graphene, while CNTs exhibit low gauge factor (GF) values, and other elastomers showed low efficiency [8,15–18]. Therefore, the choice of STCEs materials directly affects the durability of the sensors and the signal stability under deformation. For accurate and prolonged strain sensing, these materials must combine mechanical flexibility, stretchability, and sustained high electrical conductivity [6,18,19]. Among the various materials explored for STCEs, silver nanowires (AgNWs) and polyurethane (PU) have emerged as promising candidates owing to their excellent conductivity and moderate optical transparency. In particular, AgNWs are well known for their high conductivities, optical transparency, and excellent biocompatibility, all of which satisfy the aforementioned requirements for STCEs. Moreover, the excellent solution processability of AgNWs facilitates mass production and large-scale fabrication of wearable electronics [20–26]. The potential in AgNWs for transparent and flexible devices has been extensively reported [27–36]. Despite their high performance, AgNWs are limited by their brittle nature, which degrades their electrical performance under repeated stretching and bending. This brittleness leads to the detachment of nanowires from the substrate or the disconnection of the AgNW network, significantly increasing the electrical resistance of the sensor. Amjadi et al. [18] reported that repeated strains increased the number of detached AgNWs, thereby increasing the electrical resistance of the film. Detachment of AgNWs from the substrate is a critical issue in most next-generation applications, particularly strain sensors. Meanwhile, PU – a highly flexible and durable elastomer – offers the necessary mechanical resilience to withstand repeated deformation, making it an ideal candidate for enhancing the mechanical stability of AgNW-based electrodes [37,38]. PU also has the benefit of biocompatibility, which is particularly important for applications involving direct contact with the skin for prolonged periods. Its excellent stretchability and toughness allow it to retain its mechanical properties even after repeated stretching, bending, and compression [37–42]. Although the potential of both AgNWs and PU materials in stretchable and wearable electronics is well established, comprehensive studies on bilayer composites with varying AgNW-PU contents and their applications remain limited.

In this study, we present highly conductive and stretchable AgNW-PU composite electrodes with a bilayer structure designed for use in heater devices, strain sensors, and interconnectors. Although Tian et al. [43] demonstrated highly stretchable e-textiles using screen-printed fractal silver dendrites, their method involves complex processes including precise patterning and additional waterproof coatings, significantly increasing fabrication complexity and cost. In comparison, our one-step bar-coating technique enables direct fabrication of AgNW-PU bilayer electrodes with tunable properties simply by adjusting the Mayer bar number and coating speed, requiring no post-patterning or special treatments. In this configuration, the bottom layer, which is rich in AgNWs, primarily serves as the conductive pathway, whereas the top PU-rich layer provides mechanical reinforcement and elastic protection. This design significantly improves the electrode reliability under repeated deformation. The benefits of this bilayer structure were confirmed through mechanical stress testing using a custom-built bending tester, particularly for strain-sensor applications. The effects of PU blending and bilayer deposition were analyzed using scanning electron microscopy (SEM) and through performance evaluation of the heaters, sensors, and interconnectors. Owing to the high conductivity of AgNWs and the excellent stretchability of PU, the resulting bilayer composite electrode achieved an optimal balance between electrical performance and mechanical durability. These qualities render it particularly suitable for wearable electronics, which require both flexibility and long-term reliability. The effectiveness and practical viability of the AgNW-PU hybrid electrodes were thoroughly validated, underscoring their potential for various stretchable and wearable sensor applications.

## 2. Experimental details

### 2.1. Bar coating of AgNW-PU composite

**Figure 1.**
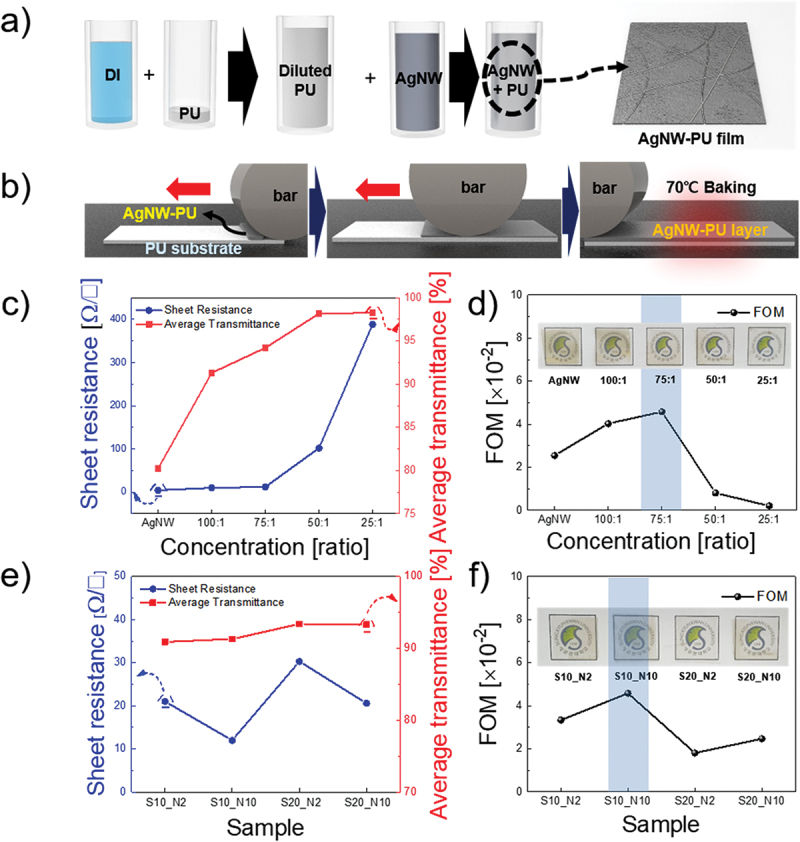
(a) Schematic of the synthesis of PU mixed AgNW solutions. (b) Schematic of the bar-coating process. (c) Sheet resistance and average transmittance with respect to the solution concentration. (d) FOM values respect to the solution concentration. Photograph of each film on top. (e) Sheet resistance and average transmittance with respect to the bar-coating speed and bar number. (f) FOM values with respect to the bar-coating speed and bar number. Photograph of each film on top.

For bar coating of the AgNW-PU composite film, water-based PU (U3251, Alberdingk Boley) was diluted with deionized water again at a ratio of 1:10, as shown in Figure 1(a). For optimization, water-based AgNW solutions (length, 25 μm; diameter, 25 nm; Flexio Co. Ltd) were mixed with diluted PU with variety of ratios-100:1, 75:1, 50:1 and 25:1. Figure 1(b) shows schematics of the bar-coating process. The mixed solution was dropped onto a PU substrate (2 × 5 cm^2^) and fixed on a knife (Mayer Bar). The coating device (KP-3000vH, Keepae) was heated to 70 °C. To optimize the bar-coating process, we applied four combinations of bar-coating speeds of 10 and 20 mm/s. The optimized coating speed was 10 mm/s, and bar No. 10 was used. After bar coating, the samples were heated for 10 min above 70 °C for solvent vaporization. After the solvents were fully vaporized, the AgNW-PU solution was dropped again, and an identical process was repeated for double-layer processing.

### 2.2. Fabrication of heater devices, strain sensors and LED interconnectors

Flexible and stretchable heaters were fabricated by cutting the bilayer – coated substrates into small pieces (2 × 4 cm^2^) for precise measurements. First, Ag paste was applied to both ends of the samples over an area of 0.5 × 4 cm^2^ to form contact points. Then, Cu tape of identical size was attached above the Ag paste to improve the contact with the power supply (OPS-3010, ODA Technologies). The temperature of the stretchable heater was measured using a hot-chuck controller (MST-1000 H, MSTECH). For the strain sensor, Ag paste and Cu tape were attached in the same manner as the heater device, followed by attachment to the index finger. The Cu wires connected to a digital multimeter (DMM, 34470A, Keysight) were placed above the Cu tape and fixed by covering them again. The entire electrode was then tightly fixed to the finger with transparent tape wound only above the Cu-tape parts, leaving the main electrode parts undisturbed. The slight pressure exerted by fingers can be regarded as a secondary disturbance factor in the real wearing environment and has been verified by various studies as an acceptable level [44,45]. After fixation, mechanical strain of the electrode was induced by repeated bending and stretching of the fingers, which caused the electrode to move along the skin. Mechanical deformation changes the electrode length, affecting its resistance. By recording the resistance value at 1-s intervals, the dynamic response of the electrode to finger movements was monitored to evaluate its strain sensitivity and stability in an actual wearable environment.

To further demonstrate the practical conductive function of the developed stretchable interconnect, a simple light-emitting diode (LED) circuit model was constructed. The circuit used Cu-tape connectors and deliberately reserved an open area to simulate a situation in which stretchable interconnections are required in actual devices. The prepared double-layer stretchable electrode was inserted into the disconnected part of the circuit to serve as a bridge. When the electrode was correctly inserted, the current flowed through the electrode, lighting the LED, which visually verified that the electrode could maintain good conductivity and connection reliability even when stretched. This experiment demonstrated the excellent mechanical flexibility and stable electrical performance of the electrode, confirming its applicability to flexible electronic devices, wearable sensors, and other fields.

### 2.3. Characterization

To systematically evaluate the performance of the fabricated stretchable bilayer electrodes, we employed multiple characterization techniques for electrical, optical, and structural analyses. The electrical properties were measured using a four-point probe system (FPP-HS8, DASOL ENG) to ensure the accurate elimination of contact-resistance interference from the test results. The optical properties were tested using an ultraviolet – visible spectrometer (V670, Jasco) in the wavelength range of 400–800 nm to obtain the transmittance curve of the electrode and assess its applicability to flexible optoelectronic devices. Surface and microstructural analyses were conducted using field-emission SEM (JSM-7600F, JEOL). High-resolution imaging of the distribution, crosslinking density, and surface morphology of the AgNW network on the PU substrate was performed to confirm the structural uniformity and continuity of the material. To investigate the mechanical and electrical stability of the electrode under strain conditions, a bending machine (Junil-Tech) was used to apply stress and simultaneously measure the strain changes. A bending test was performed in the laboratory using a self-made dual-mode inner/outer bending test platform to simulate the dynamic deformation process of the electrode in actual wearable environments. The inner bending test mainly evaluates the performance changes in the electrode under compressive stress, whereas the outer bending test focuses on its stability under tensile stress.

## 3. Results and discussion

To identify the optimal mixing ratio of the AgNW-PU solution, the electrical and optical properties of the AgNW-PU bilayer electrodes were systematically evaluated as a function of the mixing ratio. The ideal composition balances conductivity, transparency, and mechanical stability, which are critical factors for achieving high-performance, stretchable, and flexible electronics. Figure 1(c) presents the relationship between the sheet resistance and average transmittance for various AgNW-to-PU ratios. As the proportion of nonconductive PU increased, the sheet resistance of the AgNW-PU composite increased markedly. The pure AgNW electrode exhibited a low sheet resistance of 4.32 Ω/sq, whereas the composite with a 25:1 AgNW-to-PU ratio had a significantly higher resistance of 388 Ω/sq. This increase in resistance was attributed to the insulating nature of the PU matrix, which disrupted the percolation network of the AgNWs, limiting the charge-transport pathways and enhancing electron scattering. Meanwhile, the average transmittance of the AgNW-PU composites in the wavelength range of 400–800 nm increased with the PU content. The pure AgNW electrode exhibited an average visible transmittance of 80.23%, whereas the addition of PU increased the transmittance to 98.25% at a 25:1 AgNW/PU mixing ratio. This trend underscores the inherent tradeoff between sheet resistance and optical transmittance in AgNW-PU composite electrodes, highlighting the challenge of achieving both high conductivity and high transparency in the development of transparent electrodes. To determine the optimal AgNW-PU ratio that balances electrical conductivity and optical transparency, the figure of merit (FOM) was calculated. The FOM quantitatively assesses the electrode performance by accounting for the tradeoff between sheet resistance and optical transmittance. The equation used for FOM calculation is as follows:

(1) $$\mathrm{FOM}=10^{3}\frac{{T_{\mathrm{av}}}^{10}}{R_{s}}$$

Where $T_{\mathrm{av}}$ represents average transmittance and $R_{s}$ denotes sheet resistance. The calculated FOM values are shown in Figure 1(d), with the 75:1 AgNW-PU solution exhibiting the highest FOM value of 4.58. According to this result, a 75:1 AgNW-PU ratio was selected for optimization of the bar-coating speed and film thickness. Figure 1(e) shows the optimization process for the bar-coating parameters using the optimized solution. The bar number, which was determined by the diameter of the coating bar and the resulting wrinkle-like periodic hollows, influenced the final layer thickness. For bar No. 2, the deposited layer thickness was 4.57 μm, whereas bars No. 10, 30, and 50 produced layer thicknesses of 22, 68, and 114.3 μm, respectively (Table S 1). The coating speed primarily affects the uniformity of the film, with higher speeds often resulting in non-uniform coatings. Therefore, two critical parameters – film thickness, which affects the mechanical properties, and coating speed, which governs the uniformity – were systematically optimized. Bar numbers exceeding 10 yielded a layer thickness of 68.6 μm in a single deposition, necessitating bilayer deposition to achieve a target thickness of 137.2 μm. Thus, bars larger than No. 10 were excluded from further analysis. Additionally, coating speeds exceeding 20 mm/s produced insufficient coatings, which were eliminated from the optimization process. Thus, four combinations of the coating speed and bar number were selected for further analysis: bar-coating speeds of 10 mm/s with bar No. 2 (S10_N2), 20 mm/s with bar No. 2 (S20_N2), 10 mm/s with bar No. 10 (S10_N10), and 20 mm/s with bar No. 10 (S20_N10). Figure 1(e) shows the sheet resistance and average transmittance for each combination. Among the four combinations, S10_N10 exhibited the lowest sheet resistance (12 Ω/square) because of its low coating speed (10 mm/s) and thicker film (bar No. 10), ensuring a uniform AgNW network. S20_N10 (20.6 Ω/square) and S10_N2 (21 Ω/square) had slightly higher resistance owing to either a higher coating speed (20 mm/s) or thinner film (bar No. 2), reducing conductive pathways. S20_N2 exhibited the highest resistance (30.3 Ω/square), as the high speed and thin film limited AgNW connectivity.

The average transmittance remained relatively consistent across the samples, with S20_N2 exhibiting the highest value (93.4%) because of its thinner film, whereas S10_N2 exhibited the lowest value (90.91%) because of its denser AgNW network. Among the tested conditions, S10_N10 was the optimal choice, exhibiting the best balance between conductivity and transparency. To further assess the performance, the FOM was recalculated, which confirmed that a coating speed of 10 mm/s with bar No. 10 yielded the highest FOM, as shown in Figure 1(f). Figure S1 presents the FOM for all AgNW concentrations (100:1, 75:1, 50:1, and 25:1) under four fabrication conditions (S10_N2, S20_N2, S10_N10, and S20_N10). Across all solution ratios, S10_N10 consistently produced the highest FOM values, which was attributed to the slower coating allowing uniform AgNW network formation and the larger bar promoting sufficient film thickness for robust conductive pathways. Among the tested compositions, the 75:1 AgNW-PU solution exhibited the highest FOM, likely because of the optimal balance between conductivity and transparency. A higher PU content improved film uniformity and adhesion while preserving adequate AgNW connectivity for efficient charge transport. These results confirmed that the 75:1 AgNW-PU electrode provides the best tradeoff between electrical and optical performance; thus, it was selected for further processing.

**Figure 2.**
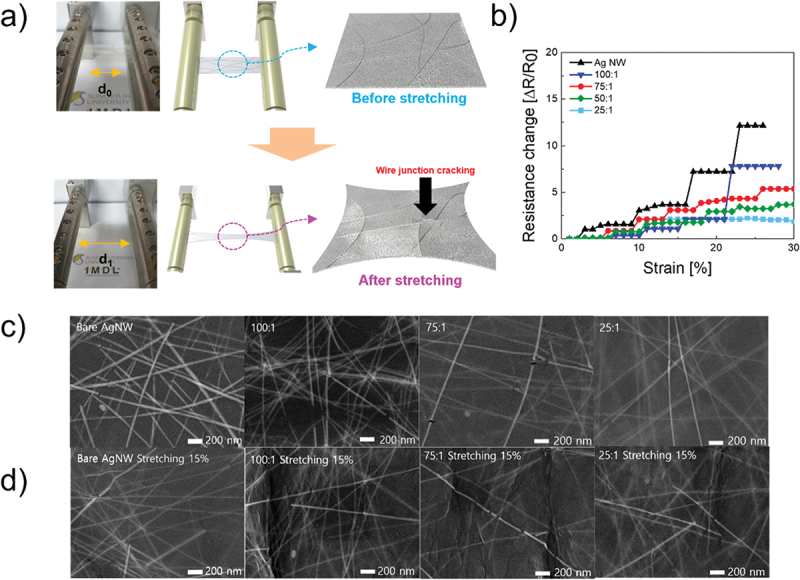
(a) Photograph of the stretching machine and schematic of the stretching process. Before stretching is shown above, and after stretching is shown below. (b) Resistance change of the single layer with respect to strain. (c) SEM images of the single layer before stretching. (d) SEM images of the single layer after stretching.

PU formed a protective coating around the periphery of the AgNWs, stabilizing the inter-nanowire connections and mitigating the slippage or separation of AgNWs under external mechanical forces (Figures S2A and S2B). A photograph of the stretching machine and schematic of the stretching process are shown in Figure 2(a). Figure 2(b) shows the variation in the resistance of the AgNW-PU composite films with different PU concentrations under an applied strain. As the PU concentration increased from pure AgNWs to a 25:1 AgNW-PU ratio, the slope of the change in resistance became more gradual, indicating enhanced mechanical stability. The pure AgNW films and 100:1 composites exhibited a sharp increase in resistance at approximately 15% strain and failed before reaching 30% strain, demonstrating poor stretchability. Figures 2(c,d) present SEM images of the AgNW-PU composite films before and after 15% stretching, respectively. Before stretching, the PU matrix effectively wrapped and filled the gaps between the AgNWs, enhancing both the mechanical stability and electrical conductivity of the network (Figure S2C). However, higher PU concentrations led to a more isolated AgNW network, which reduced the electrical connectivity and significantly increased the resistance. The PU matrix also influences the film morphology by modulating the distribution and aggregation of AgNWs, affecting the electrical and optical properties of the composite. After stretching, the buffering effect of PU mitigated the separation of the AgNWs, improving the durability and stability of the film through elastic rebound (Figure S2D). In contrast, pure AgNW films and composites with ratios of 100:1 and 75:1 exhibited notable cracking at the nanowire junctions and within the film after deformation. The bare AgNW sample without PU encapsulation exhibits significant network rupture, nanowire detachment, and aggregation after 15% uniaxial stretching, indicating a lack of effective mechanical support. When the PU:AgNW mass ratio is 100:1, the network structure shows slight improvement; however, numerous cracks and voids are still observed. With the increase in PU concentration to 75:1, the adhesion between the nanowires and the polymer matrix is enhanced, leading to a reduction in both aggregation and void formation. At the highest concentration of 25:1, the composite film remains relatively intact after stretching, with only a few minor cracks, and the continuity of the AgNW network is significantly improved. In summary, as the PU concentration increases, the structural stability and crack resistance of the composite film are improved, which is attributed to the stress-buffering effect provided by the PU matrix. This improvement was attributed to the stretchable PU matrix, which was uniformly distributed and strongly adhered to the AgNWs, providing mechanical support to prevent nanowire deformation. In the absence of PU, AgNWs experienced a rapid increase in resistance under strain owing to nanowire separation during substrate deformation, which reduced the number of conductive pathways [46]. However, owing to the significant change in resistance observed in single-layer electrodes under strain, the development of a bilayer structure is necessary. In bilayer electrodes, the upper layer primarily governs the mechanical properties, whereas the lower layer determines the electrical performance [47]. Electrically, the 100:1 AgNW-PU solution ratio was found to be optimal, exhibiting the lowest sheet resistance while maintaining a moderate strain tolerance. Mechanically, the 25:1 AgNW-PU solution ratio demonstrated superior durability while preserving electrical conductivity. Thus, a bilayer structure comprising a 25:1 AgNW-PU top layer and a 100:1 bottom layer is proposed to achieve the best balance of properties. Several reference electrodes were fabricated and tested to further explore the effects of different layer combinations. A 100:1/AgNW bilayer was prepared to assess the influence of the PU concentration when using the same bottom layer (100:1). A 100:1/100:1 bilayer was constructed to investigate the effects of the absence of a mechanically reinforced top layer. A 25:1/100:1 structure was fabricated to verify the hypothesis that the top layer predominantly affects the mechanical performance, whereas the bottom layer dictates the electrical conductivity. Additionally, a 25:1/25:1 bilayer was evaluated to compare its electrical performance with that of a 100:1/25:1 electrode. These systematic experiments were designed to optimize and clarify the tradeoff between the mechanical robustness and electrical conductivity of the AgNW-PU composite films.

**Figure 3.**
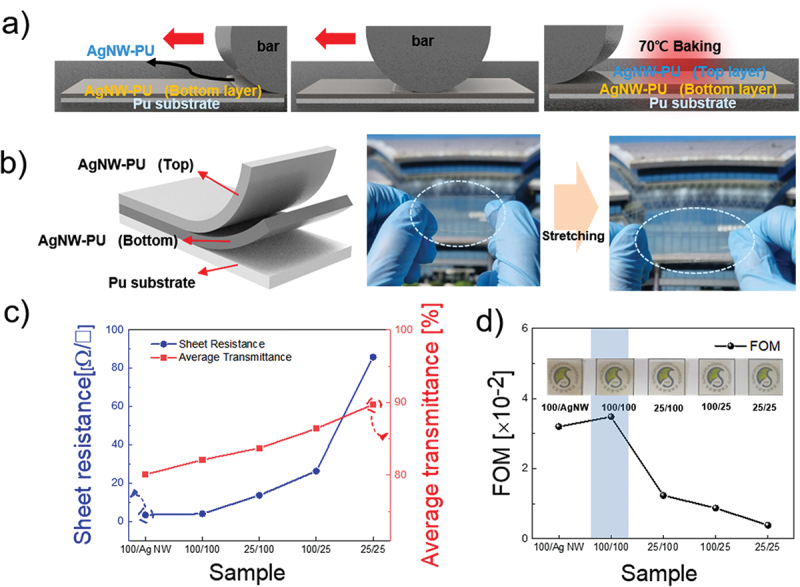
(a) Schematic of the bilayer bar-coating process. (b) Structure of the bilayer film and photograph of actual stretching. (c) Sheet resistance and average transmittance of bilayer electrodes. (d) FOM values of bilayer electrodes.

Figures 3(a,b) show a schematic of the bilayer bar-coating process and the structure of the bilayer film with actual stretching photographs. Figure 3(c) presents the sheet resistance and average transmittance of the various bilayer AgNW-PU electrodes. The 100:1/AgNW and 100:1/100:1 samples exhibited low sheet resistance of 3.39 and 3.99 Ω/square and low transmittance of 80.08% and 82.1%, respectively. This behavior was attributed to the limited presence of PU in the structure, which initially restricted light transmission. As the PU concentration increased in the 25:1/100:1 and 100:1/25:1 bilayer electrodes, the sheet resistance correspondingly increased to 13.6 and 26.3 Ω/square, respectively, accompanied by increases in optical transmittance to 83.73% and 86.4%. The 25:1/25:1 bilayer exhibited substantial increases in both sheet resistance and average transmittance, reaching 85.8 Ω/square and 89.73%, respectively. The corresponding FOMs are shown in Figure 3(d). The 100:1/100:1 electrode exhibited the highest FOM value; however, it is important to recognize that the FOM primarily captures the electrical and optical properties without accounting for mechanical stability.

**Figure 4.**
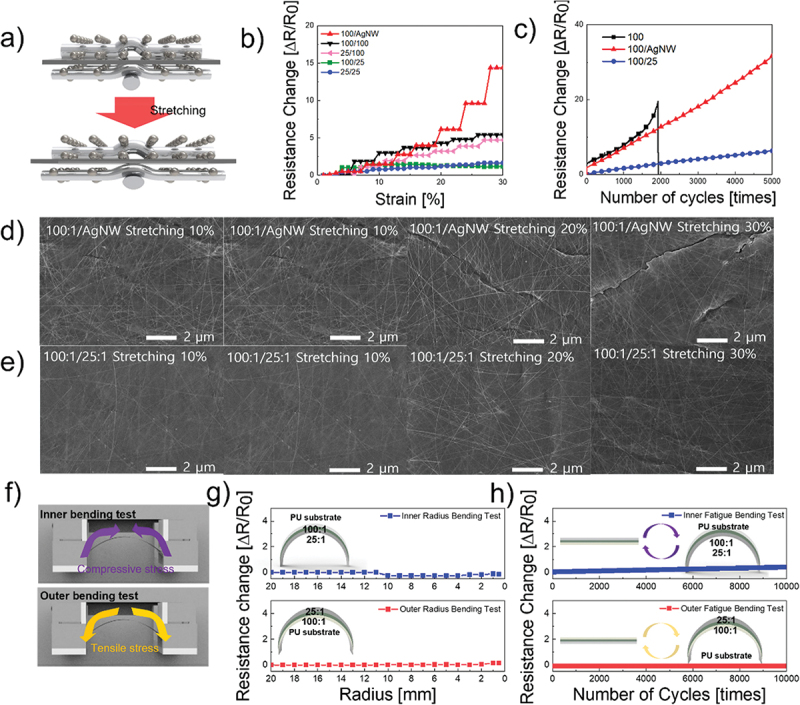
(a) Schematic of the double layer during stretching. (b) Resistance change of double layers with respect to strain. (c) Resistance changes of 100:1, 100:1/AgNW, and 100:1/25:1 during 5000 cycles of 15% stretching. (d) SEM images of double layers before stretching. (e) SEM images of double layers after stretching. (f) Schematic of the inner/outer bending test. (g) Inner/outer bending test of 100:1/25:1 for different bending radii. (h) Inner/outer fatigue bending test of 100:1/25:1 for 10,000 cycles.

Figure 4(a) illustrates the bilayer stretching process. Considering mechanical properties, such as stretching, the results in Figure 4(b) indicate that the optimized electrode configuration is 100:1/25:1, as expected. Compared with single-layer stretching, the bilayer structure offers a better balance between mechanical strength and electrical performance. The top layer provides mechanical support, whereas the bottom layer ensures conductivity, making the bilayer more suitable for higher strains than the single-layer configuration. This outcome aligns with the initial hypothesis that a high-conductivity bottom layer (100:1) combined with a mechanically resilient top layer (25:1) offers the best balance among electrical performance, optical transparency, and mechanical durability for wearable strain sensors. When the bottom-layer concentration was fixed at 100:1, the strain resistivity increased as the PU concentration in the top layer increased from 100:1/AgNW to 100:1/100:1 and finally to 100:1/25:1. This trend mirrors that observed in the single layers. Similarly, when the bottom layer was deposited at a 25:1 ratio, the strain resistivity increased as the PU concentration in the top layer increased. Although the 25:1/25:1 bilayer may appear to be a promising option according to the figure, its initial sheet resistance of 85.8 Ω/square was considerably higher than that of the 100:1/25:1 bilayer, which has a resistance of 26.3 Ω/square. This resulted in a lower overall resistance change owing to the higher starting resistance. Figure 4(c) shows the resistance change over 5000 cycles of 15% stretching. The 100:1 single-layer electrode failed within 2000 cycles, whereas the 100:1/AgNW bilayer electrode withstood up to 5000 cycles but exhibited a significant resistance change. In contrast, the 100:1/25:1 bilayer electrode endured 5000 cycles with minimal resistance changes. This highlights the importance of both the passivation and top layers in improving mechanical performance. These effects are further supported by the SEM images shown in Figures 4(d,e). Figure 4(d) shows the stretching behavior of the 100:1/AgNW double layer from 10% to 30%. The film was critically damaged at a strain of 20% and nearly tore at 30%. In contrast, Figure 4(e) shows a 100:1/25:1 bilayer stretching from 10% to 30% with only minor damage observed, confirming the superior mechanical stability of this configuration. Figures 4(g,h) present the results of the inner and outer bending and fatigue bending tests on the 100:1/25:1 bilayer electrode. As shown, the electrode exhibited both stretching and bending. Even under a critical bending radius of 0.5 mm, the change in resistance was minimal. After 10, 000 cycles of inner and outer fatigue bending tests, no significant resistance changes were observed, indicating excellent durability.

**Figure 5.**
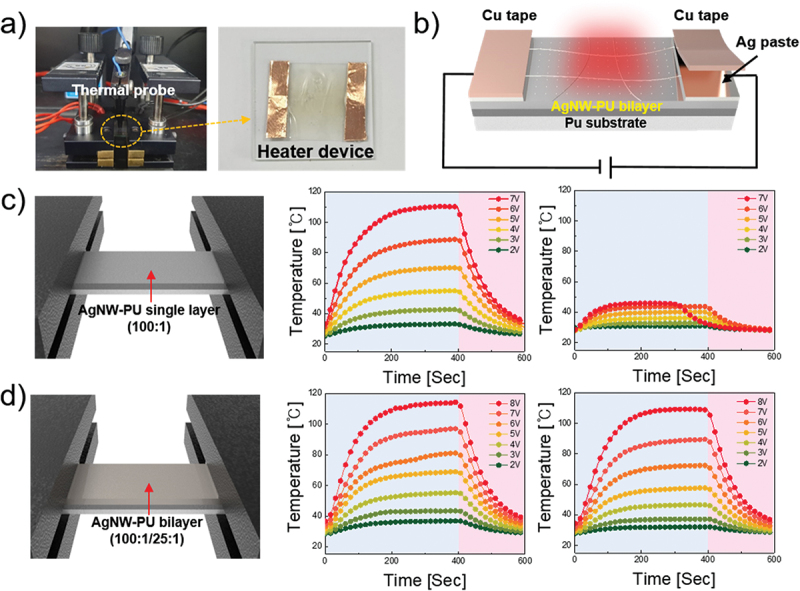
(a) Photograph of the jig and thermal probe measuring temperature generation and a photograph of the heater device. (b) Schematic of depositing Ag paste and Cu tape above the AgNW-PU film. (c) Schematic of measuring the temperature generation of the 100:1 single layer and the results before and after stretching. Each voltage was applied for 400 s, followed by 200 s of cooling. (d) Schematic of measuring the temperature generation of the 100:1/25:1 double layer and the results before and after stretching. Each voltage was applied for 400 s, followed by 200 s of cooling.

A heater device was designed to evaluate the performance of the optimized electrode for practical applications. Figures 5(a,b) show a schematic and the actual setup of the heater device, respectively. Ag paste was applied to the ends of the electrode to serve as contact points, and Cu tape was added to ensure better electrical contact with the power supply. The temperature generated by the heater was measured using a thermal probe placed at the center of the electrode. Because electrical properties are critical for heater devices, the 100:1 single-layer electrode was used as a reference, and the 100:1/25:1 bilayer electrode was tested to verify its superior performance. The temperature generated in the heater device was calculated using the following equation [48,49]:

(2) $$\Delta Q_{s}=\frac{V^{2}}{R_{s}}\Delta t=Q_{\mathrm{conv}}=h_{\mathrm{conv}}A_{\mathrm{conv}}\left(T_{s}-T_{i}\right)$$

**Figure 6.**
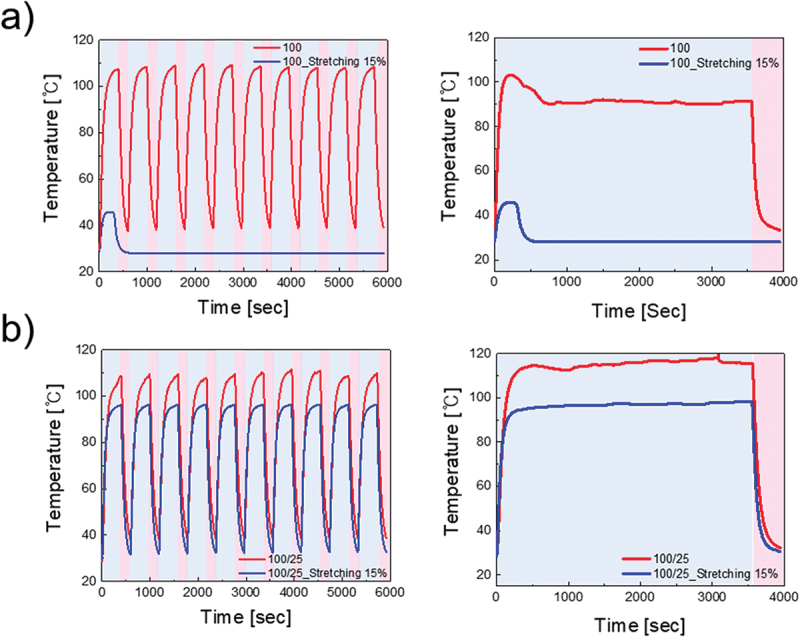
(a) Heater fatigue test (left) and stability test (right) for the 100:1 single layer before and after stretching. (b) Heater fatigue test (left) and stability test (right) for the 100:1/25:1 double layer before and after stretching.

Where $\Delta Q_{s}$ represents the generated temperature, V represents the input voltage, and *t* represents the heating time. $Q_{\mathrm{conv}}$ represents heat loss due to convection in air, $h_{\mathrm{conv}}$ is the convective heat-transfer coefficient, $A_{\mathrm{conv}}$ represents the surface area, $T_{s}$ represents the saturation temperature, and $T_{i}$ represents the initial temperature. The heating time *t* was kept constant for both the 100:1 single layer and the 100:1/25:1 bilayer. When the same voltage is applied, the equation is simplified to show that the generated temperature is inversely proportional to the sheet resistance. As indicated by Figures 5(c,d), at a voltage of 7 V, the 100:1 single-layer electrode generated a temperature above 110 °C, whereas the 100:1/25:1 bilayer electrode generated approximately 100 °C. Because the generated temperature was inversely proportional to the sheet resistance, the 100:1 electrode exhibited a lower sheet resistance than the 100:1/25:1 bilayer, as confirmed experimentally. Therefore, the sheet resistances can be effectively compared according to the temperature generation. Figure S3 presents the temperatures generated by the 100:1 and 100:1/25:1 electrodes at different voltages before and after stretching. With optimization of the current flow, rational distribution of the resistance, and mechanical support, the 100:1/25:1 bilayer electrodes maintained a lower temperature generation rate during stretching and generated higher temperatures after stretching, while offering greater mechanical stability. Thus, the bilayer structure is superior to single-layer electrodes for practical applications. However, after a strain of 15% was applied, the heat-generation phase shifted significantly. For the 100:1 single layer, the heater property was almost eliminated after stretching, with the maximum generated temperature hardly exceeding 40 °C, and the layer broke at 7 V. In contrast, the 100:1/25:1 double layer exhibited nearly identical performance after stretching. These results were even more pronounced in the heater fatigue and stability tests, as shown in Figures 6(a,b). Furthermore, the 100:1 single-layer electrode exhibited uniform and high-quality heating performance during the fatigue test. However, after 15% stretching, it failed to withstand a single cycle and lost its heating function. In the stability test, after 7 V was applied for 350 s, the 100:1 single-layer electrode could not maintain a stable temperature and only reached approximately 90 °C. In contrast, the 100:1/25:1 bilayer electrode maintained consistent temperature generation even after stretching, with only a 15 °C reduction in the maximum temperature after 10 fatigue-test cycles. Stability was also preserved after 15% stretching, with heat generation remaining above 95 °C. With the incorporation of a 25:1 layer as a passivation layer, the electrode exhibited significant improvements in its resistance to mechanical stress. This indicates that the bilayer structure significantly enhances both the durability and stability, making it a superior choice for flexible and stretchable heater applications.

**Figure 7.**
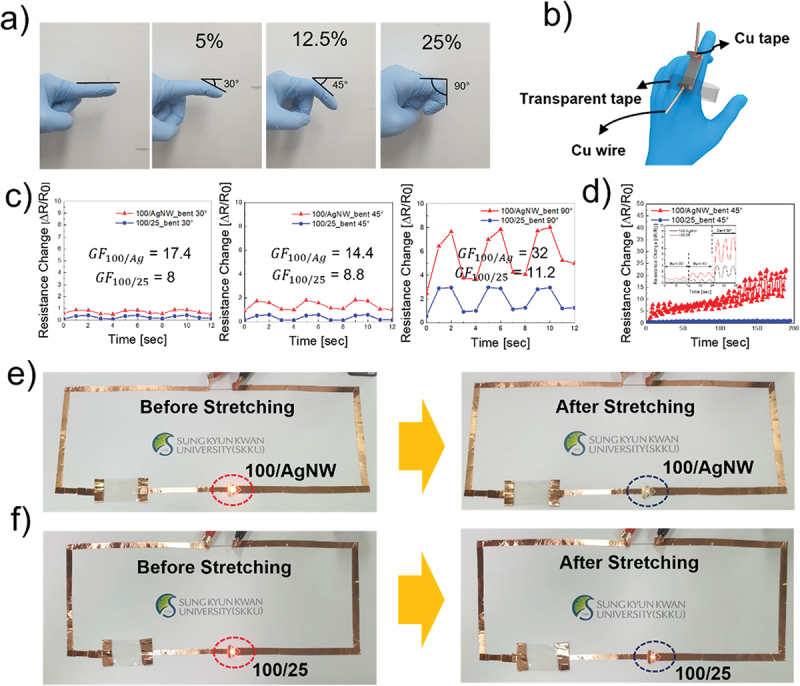
(a) Photograph of bending degree during strain-sensor test. (b) Schematic of fixing strain sensor on the finger. (c) Resistance change of 100:1/AgNW and 100:1/25:1 for bending 30°, 45°, and 90° for 12 s. (d) Resistance change of 100:1/AgNW and 100:1/25:1 for bending 45° for 200 s. (the illustration shows the continuous and complete sensing curves of the two electrodes under different strains). (e) Photograph of interconnector with 100:1/AgNW electrode before stretching (left) and after stretching (right). (f) Photograph of interconnector with 100:1/25:1 electrode before stretching (left) and after stretching (right).

A strain-sensor test was conducted to assess the mechanical stability and electrical response. Figures 7(a,b) illustrate the strain-sensor testing process. Similar to the fabrication of the heater device, Ag paste was applied to both ends of the film, and Cu tape was placed on top to improve the electrical contact. The Cu wires were then connected to Cu tape and attached to a DMM. The two ends were fixed to a finger using transparent tape. Figure 7(c) presents the resistance changes at various strain levels between the 100:1/AgNW and 100:1/25:1 bilayers. As the 100:1 single-layer electrode exhibited poor performance under stretching, the reference electrode was modified into a 100:1/AgNW bilayer. A 30° bend caused a resistance change of 0.87 for the 100:1/AgNW layer, whereas the 100:1/25:1 bilayer exhibited a smaller resistance change of 0.4. As the bending angle increased, the resistance-change difference between the two electrodes increased. At a 45° bend, the 100:1/AgNW layer exhibited a resistance change of 1.8, while that of the 100:1/25:1 bilayer was 1.1. At 90° bending, the 100:1/AgNW layer experienced a resistance change of 8, whereas that of the 100:1/25:1 bilayer was only 2.8. In strain sensors, the ratio of the relative change in resistance to the strain is expressed as the GF, which is calculated as

(3) $$\mathrm{GF}=\frac{\Delta R/R_{0}}{\varepsilon }=\frac{R-R_{0}}{R_{0}\times \varepsilon }$$

Where ε represents strain applied to the sensor, $R_{0}$ represents the initial resistance, and R represents the final resistance at a strain of ε [42,48]. The strain (ε) can be determined by the change in sensor length, and it was calculated as 5%, 12.5%, and 25% for bending angles of 30°, 45°, and 90°, respectively. The GFs for the 100:1/AgNW bilayer were calculated to be 17.4 at 30°, 14.4 at 45°, and 32 at 90°. The 100:1/25:1 bilayer exhibited GF values of 8 at 30°, 8.8 at 45°, and 11.2 at 100°. Initially, no significant difference was observed between the 100:1/AgNW and 100:1/25:1 electrodes. However, when bending was applied, the film experienced lateral stress, resulting in strain. This strain remained manageable at 30° bending: it was only 5%, which was tolerable for both electrodes. At a 45° bending angle, the difference in the resistance change between the two electrodes increased. When the bending angle reached 90°, resulting in 25% strain, the resistance change in the 100:1/AgNW electrode became excessive and uncontrollable. Consequently, the 100:1/25:1 bilayer was identified as the optimal electrode with regard to conductivity, transparency, and stretchability. The high stretchability and sensitivity of this strain sensor make it suitable for a wide range of applications, particularly wearable electronics and human motion detection. Simple actions, such as finger bending, induce distinct electrical-property changes, allowing precise categorization of movements owing to the sensor’s sensitivity to subtle bending variations. As shown in Figure 7(d), stability testing supported these findings. During 200 s of repeated 45° bending, the 100:1/AgNW bilayer underwent significant changes in resistance, indicating degradation. In contrast, the 100:1/25:1 bilayer maintained its structural and electrical integrity over the same period, demonstrating superior durability. This result confirmed the enhanced stability of the optimized strain sensor, which makes it highly promising for industrial applications. The illustration in Figure 7(d) also indicates that the resistance change of the 100:1/25:1 bilayer electrode at each strain is not as obvious as that of the 100:1/AgNW bilayer electrode. Figures 7(e,f) show the results of the interconnector tests. Figure 7(e) presents the LED light emissions before and after stretching of the 100:1/AgNW bilayer. The degree of light emission serves as an indicator of the electrical current flow. Because of the mechanical fragility of the 100:1/AgNW bilayer, stretching by 15% significantly damaged the electrode, significantly reducing the light emission, which was hardly visible. In contrast, the 100:1/25:1 bilayer maintained nearly the same light-emission intensity after stretching, confirming its superior mechanical and electrical stability. Figure S4 shows a photograph of the turned-off interconnector. These findings confirm that the 100:1/25:1 electrode offers excellent electrical performance even under substantial mechanical deformation.

## 4. Conclusion

We designed and optimized a highly transparent and stretchable AgNW-PU bilayer electrode that exhibited exceptional mechanical stability and electrical performance. The optimized 100:1/25:1 bilayer electrode demonstrated a low sheet resistance of 26.3 Ω/square and high transparency of 86.4%. It exhibited significant mechanical improvements, including enhanced performance in stretching, fatigue, and bending tests. While reference electrodes in the heater-device test completely lost functionality after stretching, the optimized electrode maintained its performance, with only a minor 15 °C temperature drop in heater stability tests. Furthermore, the optimized electrode demonstrated strong potential for a wide range of industrial applications by accurately distinguishing input variations such as different degrees of finger bending. The stability of the strain sensor emphasizes the robustness of this electrode for wearable and flexible electronics. Finally, interconnector tests confirmed its durability under mechanical strain, which ensures stable electrical connections. These results highlight the potential of the AgNW-PU composite bilayer electrode for applications in flexible and wearable electronics, including stretchable sensors, heaters, and interconnectors.

## Supplementary Material

Supplemental Material
