# Supplementary material for: Highly stretchable transparent Ag nanowire-polyurethane hybrid bilayer electrodes for multifunctional applications
Source: Sci Technol Adv Mater. 2025 Jul 4;26(1):2528595. doi: 10.1080/14686996.2025.2528595 (PMC12312144; doi:10.1080/14686996.2025.2528595)
Supplement: Supplemental Material [file TSTA_A_2528595_SM3205.docx]

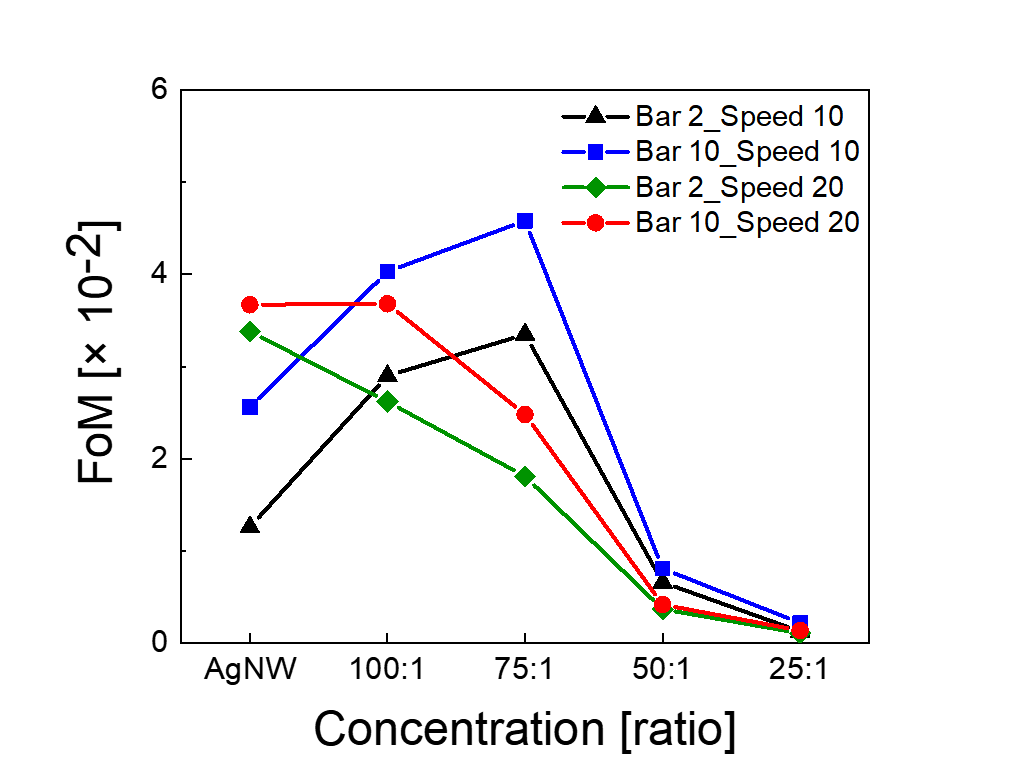


Figure S1. FOM values at various concentration ratios for four fabrication conditions.


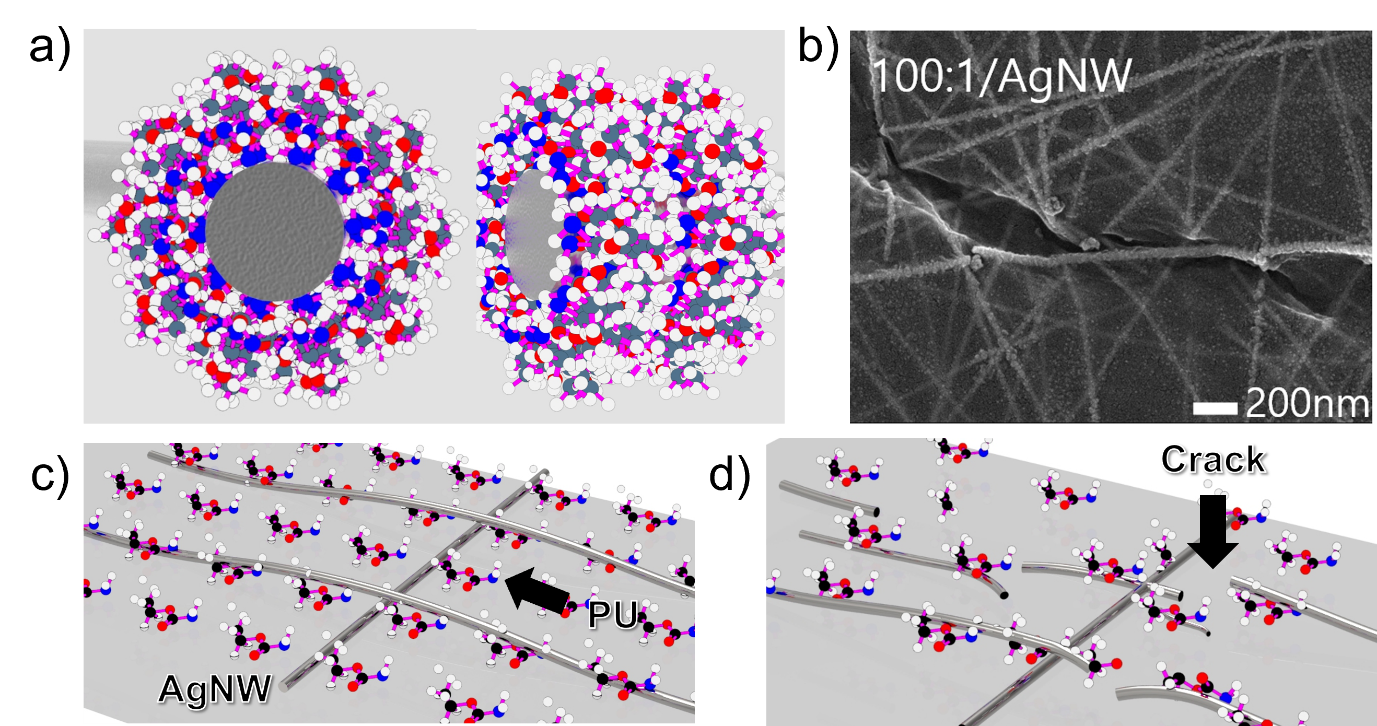


Figure S2. A) Schematic of PU supporting the AgNW. B) SEM images of PU supporting AgNWs. C) Schematic of the AgNW and PU mixed films before stretching. D) Schematic of the AgNW and PU mixed films after stretching.


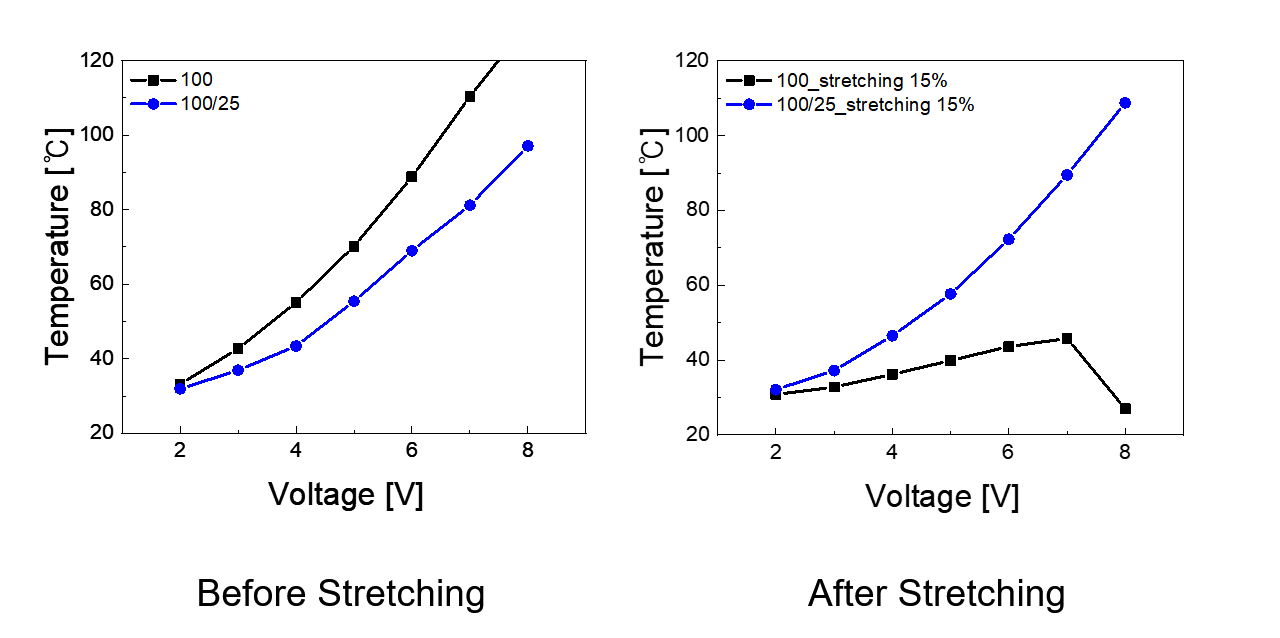


Figure S3. Temperature generation at 100:1, 100:1, and 25:1 for each voltage.


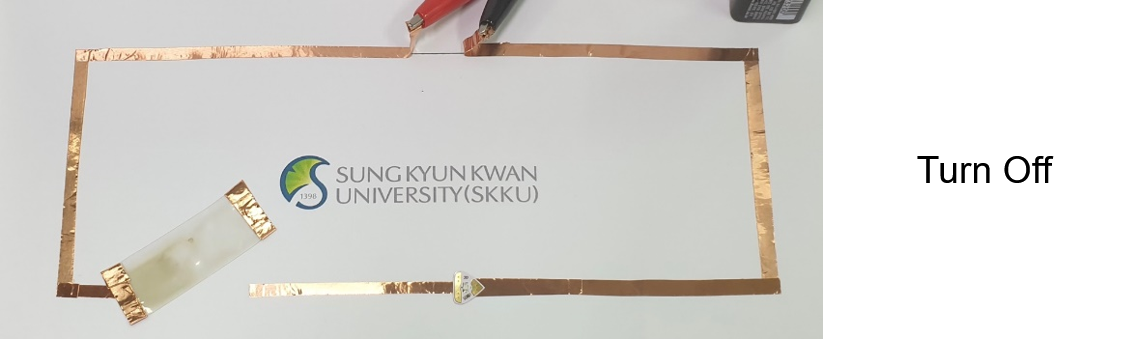


Figure S4. Photograph of the interconnector when turned off.
